# Mercury Dynamics and Bioaccumulation Risk Assessment in Three Gold Mining-Impacted Amazon River Basins

**DOI:** 10.3390/toxics12080599

**Published:** 2024-08-18

**Authors:** Vitor Sousa Domingues, Carlos Colmenero, Maria Vinograd, Marcelo Oliveira-da-Costa, Rodrigo Balbueno

**Affiliations:** 1 Brazilian Institute of Environment and Renewable Natural Resources, Brasília 70818-900, Brazil; 2 CV Consulting and Analysis, Pedro Leopoldo 33250-075, Brazil; colmenero.carlos@gmail.com; 3 Centre for Environmental Policy, Imperial College London, London SW7 1NE, UK; maria.vinograd@imperial.ac.uk; 4 World Wide Fund for Nature (WWF-Brazil), Brasília 70377-540, Brazil; marcelo@wwf.org.br (M.O.-d.-C.); rodrigobalbueno@wwf.org.br (R.B.)

**Keywords:** mercury dynamics, methylmercury, ecotoxicology, health risk assessment, environment, fish, gold mining, Amazon, traditional communities

## Abstract

Mercury contamination from gold mining in the Amazon poses significant environmental and health threats to the biome and its local populations. The recent expansion of non-industrial mining areas has severely impacted territories occupied by traditional communities. To address the lack of sampling data in the region and better understand mercury dynamics, this study used the probabilistic model SERAFM to estimate the mercury distribution and bioaccumulation in fish. The analysis covered 8,259 sub-basins across three major Amazonian basins: the Branco, Tapajós and Xingu rivers. The findings revealed increasing downstream mercury levels, with notable accumulations in the main watercourses influenced by methylation processes and mining releases. The projected concentrations showed that an average of 27.47% of the sub-basins might not comply with Brazilian regulations, rising to 52.38% in the Branco and Tapajós river basins separately. The risk assessment of fish consumption based on the projections highlighted high mercury exposure levels among traditional communities, particularly indigenous populations, with an average of 49.79% facing an extremely high risk in the Branco and Tapajós river basins. This study demonstrated SERAFM’s capacity to fill information gaps in the Amazon while underscoring the need for enhanced data collection, culturally sensitive interventions and regulatory updates to mitigate mercury contamination in gold mining-affected areas.

## 1. Introduction

The extensive expansion of illegal gold mining in the Amazon has led to serious social and environmental problems, notably mercury contamination [1]. Mercury pollution severely impacts the biome’s rich biodiversity and poses significant risks to the health and survival of Amazonian communities [2]. According to MapBiomas (2023), territory where non-industrial gold mining is found increased by 77% between 2017 and 2022, expanding from 149 to 263 thousand hectares [3]. A substantial portion of this activity takes place in protected areas. Worryingly, indigenous territories have seen a 265% rise in the spatial spread of illegal gold mining, now covering 25.2 thousand hectares, an area larger than the city of Amsterdam [3]. The most affected territories include the Kayapó (13.7 thousand hectares), Munduruku (6.5 thousand hectares) and Yanomami (3.3 thousand hectares) indigenous lands, located within the Xingu, Tapajós and Branco river basins, respectively [3].

Mercury is one of the planet’s most concerning chemicals for public health [4]. Its widespread toxicity, compounded by its capacity to accumulate and disperse broadly, has raised significant global concerns [5]. The metal adversely affects the central and peripheral nervous systems in humans and other vertebrates, harms the digestive, immune, hormonal and reproductive systems and may damage the lungs, kidneys and liver [2,6]. Consequently, communities exposed to mercury experience heightened health risks, including neurological disorders, cognitive impairments and developmental deficits in children [7]. These health impacts further exacerbate the vulnerability of communities already facing socioeconomic challenges, threatening their culture and even survival [8].

In Amazonian populations, mercury contamination levels are alarmingly high, particularly among traditional communities which rely on fish as their primary protein source [9]. The metal bioaccumulates in fish and can biomagnify through food chains, resulting in elevated mercury levels in fisheries consumed by these populations, thereby increasing exposure [10].

Monitoring the metal and mitigating its impacts are crucial for managing pollution and safeguarding communities [11]. However, efforts are hampered by the vast territory, limited resources, lack of information and illegal nature of most gold mining operations, which obscure the full extent of the impacts [11]. Despite Brazil’s ratification of the Minamata Convention on Mercury, which aims to protect human health and the environment from anthropogenic mercury emissions, implementing efficient strategies remains a challenge [12]. Given the scarcity of reliable information, exploring alternative tools and technologies becomes imperative to generate sound scientific evidence which can underpin effective interventions in mercury-contaminated regions [13].

This study aims to tackle some of these challenges by estimating the mercury distribution and bioaccumulation in gold mining-impacted Amazonian basins, using a mathematical model based on data from other published studies and scientific databases. The study further assesses the risks to local communities by relating the projected mercury bioaccumulation to fish consumption patterns in traditional populations. The primary goals are to evaluate the capacity of a model to overcome the lack of monitoring data and provide insights into mercury dynamics. Therefore, these insights could inform risk management and policy strategies to mitigate the impacts of mercury contamination in the Amazon.

## 2. Materials and Methods

### 2.1. Study Area

**Figure 1:**
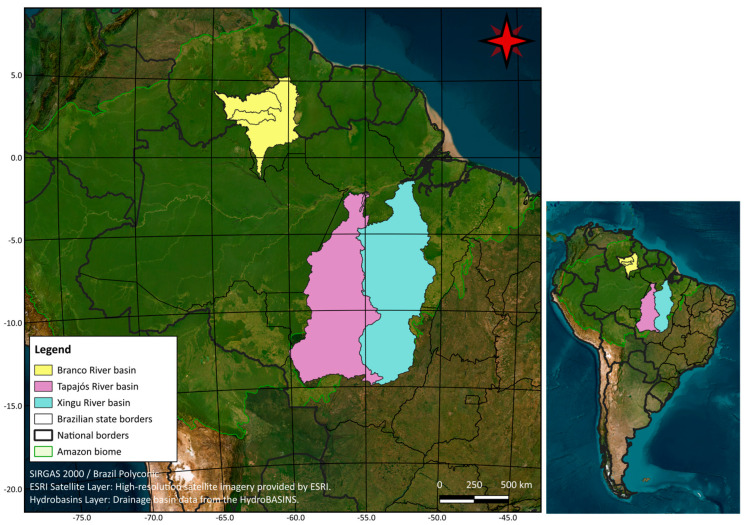
Map of the study area highlighting the three modeled river basins, including subdivisions of the Branco River basin.

This study was conducted across three key Amazonian watersheds: the Tapajós, Xingu and Branco river basins (Figure 1). Within the Branco River basin, this study specifically focused on the Mucajaí and Uraricoera watercourses. The chosen watersheds support biodiverse ecosystems and traditional communities and encompass areas severely impacted by gold mining, including the three most affected Brazilian indigenous territories [3], which motivated their selection for our analysis.

The Tapajós and Xingu river basins are among the five Amazonian basins most impacted by non-industrial gold mining activities [3]. Together, the three studied basins span approximately 119,835 thousand hectares, representing over 15% of the entire Amazon watershed and extending into the Cerrado ecosystems [14]. In addition to the most affected indigenous territories, the basins also support a plethora of other traditional communities, including riverine communities, and major cities such as Santarém, Altamira and Boa Vista.

To facilitate regional analysis, each watershed was divided into sub-basins using the 12th level vectorization proposed by the HydroBASINS database [15]. This approach allows for a more granular understanding of the environmental dynamics within each catchment area, enabling more targeted assessments.

### 2.2. SERAFM Modeling

Scenarios were projected using the SERAFM framework, a spreadsheet-based model developed by the United States Environmental Protection Agency [16]. Projections rely on data from scientific databases and literature reviews rather than direct laboratory measurements to address challenges related to mercury contamination in Amazonian basins significantly impacted by gold mining.

Zhu et al. (2018) reviewed mercury simulation models, noting SERAFM’s ability to incorporate watershed transportation processes, physicochemical transformations and bioaccumulation across trophic levels, emphasizing its detailed partitioning structure and public availability [17]. SERAFM has been successfully used to assess mercury contamination in various water bodies.

In the Peruvian Amazon, Agurto (2012) used the model to evaluate mercury contamination from gold mining activities, calibrating it with regional data and comparing the results to international standards [18]. Kim et al. (2022) also applied SERAFM to predict major mercury sources in the Arctic with a mean bias of 12% and a calibration error of 0.035 [19]. The model’s versatility is further highlighted by its application in predicting historical mercury concentrations in fish [20].

The model inputs were based on 18 parameters related to watershed characteristics to provide insights based on existing data, with the core driving inputs being land use, watercourse characteristics and water quality parameters. The parameterization was conducted while considering the characteristics of each sub-basin or generalizations when needed. All input data sources are detailed in Appendix A. Information was obtained from scientific databases or literature reviews. This approach aims to overcome the lack of direct monitoring data caused by regional obstacles, thus providing valuable insights into mercury dynamics.

Additional parameters representing background mercury concentrations were adjusted for regional accuracy, according to previous bibliographical research (Appendix A). The default rate constants related to mercury transformations remained unaltered. All the input data collected and used during the modeling process are provided in Sheet S1.

SERAFM can project three different scenarios, but only the second scenario was adopted for this study [19]. This choice was made considering that the second scenario represents environments exposed to current contamination, where mercury is still being introduced into the system [21]. The outputs on the methylmercury concentrations in fish were compiled and geospatially represented using the software QGIS 3.28.10 Firenze [22].

#### Assumptions

The modelling process assumed no migration of fish. Fish migration could affect the use of watersheds as analytical units for risk assessments. However, studies conducted in Amazon river basins have demonstrated that including migratory species in the analysis does not result in significant changes in mercury bioaccumulation indices and does not significantly affect the magnification factors between migratory and non-migratory fish species [23,24,25,26].

Although the migration of fish with elevated mercury levels could pose risks to distant communities, ecosystem dynamics, such as seasonal variations in rainfall and food availability, are likely to mitigate these effects [23,24]. Research indicates that these dynamics reduce the impact of migration on mercury cycling and distribution [23,24]. Furthermore, most fish species consumed by traditional communities in the Amazon are either sedentary or exhibit minimal migration, which reduces the influence of migration on mercury distribution within the total fish community [27,28]. Therefore, our study did not incorporate fish migration patterns into the model’s approach.

The modelling process assumed that abandoned mining sites detected by the MapBiomas database are contaminated land [3]. Although temporal variation could influence mercury bioaccumulation due to changes in gold mining activity and contamination patterns, abandoned sites typically show slow recovery and lasting impacts [29]. A temporal analysis was not conducted due to time constraints, though it could enhance our findings.

Finally, the model assumes a generalized dietary pattern, employing an average value for the entire region due to the absence of comprehensive data for each study area. Dietary preferences can vary substantially among traditional Amazonian communities. Therefore, collecting precise local dietary information could enable more tailored and accurate risk assessments.

All estimates used in this study were benchmarked against existing research in the region, with particular reference to the comprehensive literature review by Berzas Nevado (2010). This review synthesized data from 15 studies and identified a consumption pattern ranging from 5 to 13 portions of fish per week [10], aligning with the average estimates employed in our analysis.

### 2.3. Output Processing

The projection data underwent analysis guided by empirical information to constrain the SERAFM outputs within the ranges observed in the sampling studies. The first strategy was to employ available information from the Mercury Observatory [30]. The Mercury Observatory is a comprehensive platform which collates studies assessing mercury contamination in the Amazon region [30]. Established through a systematic review of the literature from 1980 to 2021, the Mercury Observatory aims to compile data on mercury contamination levels in human and fish samples [30]. Out of the three basins in this study, observation data on the platform was only available for the Tapajós River basin. These data were used to adjust the model parameters.

To account for variations in the mercury concentrations due to the trophic levels, fish species were classified as “non-piscivorous” and “piscivorous” based on their feeding habits, sourced from FishBase [31]. To ensure data integrity, incomplete records were excluded. For cases with missing mean mercury concentrations, imputation was carried out using the minimum and maximum concentration values, thereby retaining valuable data. Subsequently, the average mercury concentration and standard deviation were computed for each dataset.

To constrain SERAFM’s projections within the ranges observed in the sampling data, a 95% threshold was established based on a normalized data distribution. The 95th percentile was determined using the qnorm function, which calculates the quantile function for the normal distribution. The input parameters for qnorm included the mean and standard deviation of the imputed datasets to define the upper bound for the observed mercury concentrations. The entire analysis was performed using R software, a relevant tool for statistical computing and graphics [32].

There are no mercury concentrations in fish for the Rio Branco and Xingu basins available from the Mercury Observatory [30]. Therefore, data were processed according to studies available in the scientific literature. The studies are not available from the Mercury Observatory because they were published after the platform’s last update.

De Vasconcellos et al. (2022) analyzed 75 fish samples collected in the Branco River basin, finding average mercury concentrations of 0.116 ± 0.126 µg g^−1^ in non-piscivorous fish and 0.869 ± 0.655 µg g^−1^ in piscivorous fish [33]. Similarly, Souza-Araujo et al. (2022) analyzed 239 fish samples collected in the Xingu basin, reporting average mercury concentrations of 0.048 ± 0.027 µg g^−1^ in non-piscivorous fish and 0.360 ± 0.208 µg g^−1^ in piscivorous fish [34].

In both studies, a 95th percentile obtained from the standard deviation of the data for “piscivorous” and “non-piscivorous” fish was used to limit the maximum values of the projections and align the data more closely with observations in the respective basins [35]. All limits are shown in Table 1. Following the analysis, the outcomes, available in Sheet S2, were compiled and geospatially presented using QGIS [22].

### 2.4. Risk Assessment Methodology

#### 2.4.1. Legal Limits

The modeled concentration of mercury in fish for each sub-basin was compared to the Brazilian Regulation on Maximum Limits of Inorganic Contaminants in Foods for assessing legal compliance [36]. The guideline incorporates the Mercosur Technical Regulation on Maximum Limits of Inorganic Contaminants in Food, which updates and harmonizes the limits of contaminants among the bloc countries, aiming to protect public health [37].

#### 2.4.2. Daily Mercury Intake

The daily mercury intake was calculated by adding the modeled concentrations of metal in non-piscivorous and piscivorous fish multiplied by their respective consumption rates. The resulting sum was then divided by the average body weight, always considering distinctions between men and women, as shown in Table 2 [38].

Fish consumption in the Amazon region is notably high, especially among traditional communities, allocating them to the top consumers worldwide [33]. Within these communities, fisheries are the primary source of protein [39]. Fish consumption rates were determined based on previous studies in the region, accounting for the differences between riverine and indigenous communities.

Passos et al. (2008) estimated riverine fish consumption in the Amazon region to range between 115 and 171 g per day [40]. Risk assessments for riverine communities employed data from Passos et al. (2008), as similar findings have been reported by other researchers [10,23,33,41,42]. For indigenous communities, a study among the Mundukuru in the Tapajós river basin served as a reference, revealing that these families consume fish more than three times a week [38], with adult intakes estimated to be between 168.58 and 216.75 g per day.

The Amazon’s diverse traditional populations mean that some communities consume more or less fish and are thus more or less vulnerable to mercury contamination through this pathway [33]. Due to limited data on traditional populations, broad estimations were necessary [33]. The compiled data are shown in Table 3.

The proportion of non-piscivorous and piscivorous fish in one’s diet was differentiated. Piscivorous fish exhibit- higher concentrations of methylmercury due to their position at a higher level in the food chain. The data obtained by Berzas Nevado et al. (2010) indicate an average proportion of piscivorous fish in Amazonian diets of 45%, which was employed in the assessments [10]. Body weight data were derived from the Brazilian Census survey information [43].

#### 2.4.3. Risk Categories

**Figure 2:**
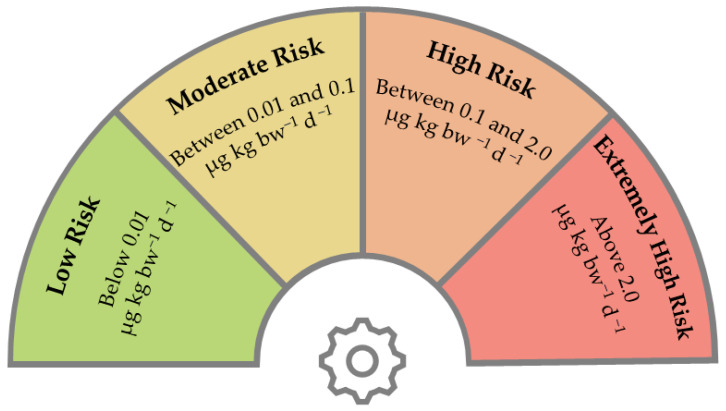
Diagram representing risk categories based on methylmercury daily intakes.

Risk assessment involved establishing risk categories by following the US EPA methodology [44]. Categories were determined according to mercury’s adverse health effects, and they are represented in Figure 2.

Categories were defined based on the effects of mercury on human health, corresponding to increasing concentrations in the body. Intervals were established, ranging from levels where no effects were observed to thresholds where damage may occur in organs affected only by higher concentrations of the metal, such as the liver and kidneys [45]. The most conservative threshold, limiting the low-risk category [46], was derived from previous epidemiological studies on mercury’s adverse effects on the nervous system, the organ most sensitive to mercury toxicity [47]. The authors set the lowest observable adverse effect level to 0.01 µg kg^−1^ day^−1^ [47].

The moderate risk category was delimited to be between 0.01 µg kg^−1^ day^−1^ and the provisional oral minimum level of 0.1 µg kg^−1^ day^−1^ defined by the American Agency for Toxic Substances and Disease Registry when assessing methylmercury toxicological effects [48]. This level is grounded in the chronic neurodevelopmental impacts associated with methylmercury exposure and establishes a limit beyond which the anticipation of chronic neurological effects arises.

For the high and extremely high risk categories, a concentration of 2 µg kg^−1^ day^−1^ was employed [49]. The threshold was determined by the WHO when considering critical effects such as nephrotoxicity when using an uncertainty factor of 100 [50]. The limit aims to prevent severe kidney and liver effects, which occur at higher exposure rates and can be fatal [46].

## 3. Results

### 3.1. Dynamics of Mercury Accumulation

In all three river basins, projections indicated a pattern of mercury accumulation which increased downstream. High concentrations were more associated with mercury methylation dynamics than the locations of gold mining sites.

In the first model, a total of 540 sub-basins were analyzed, comprising 165 in the Mucajaí river basin and 375 in the Uraricoera river basin.

The Tapajós river basin model analyzed a total of 3,791 sub-basins divided into nine regions: the Apiacás basin (113 sub-basins), Peixoto de Azevedo basin (124 sub-basins), Lower Teles Pires basin (232 sub-basins), Middle Teles Pires basin (133 sub-basins), Upper Teles Pires basin (354 sub-basins), Juruena basin (1540 sub-basins), Upper Tapajós basin (520 sub-basins), Lower Tapajós basin (362 sub-basins) and Jamanxim basin (413 sub-basins).

For the Xingu river basin, a total of 3,928 sub-basins were analyzed, divided into nine regions: the Ronuro river macrobasin (223 sub-basins), Xingu Headwaters macrobasin (328 sub-basins), Xingu and Suiá-Miçu rivers macrobasin (233 sub-basins), Manissauá-Miçu river macrobasin (221 sub-basins), Upper Xingu macrobasin (603 sub-basins), Fresco River macrobasin (338 sub-basins), Middle Xingu macrobasin (385 sub-basins), Iriri river macrobasin (1081 sub-basins) and Lower Xingu macrobasin (516 sub-basins).

**Figure 3:**
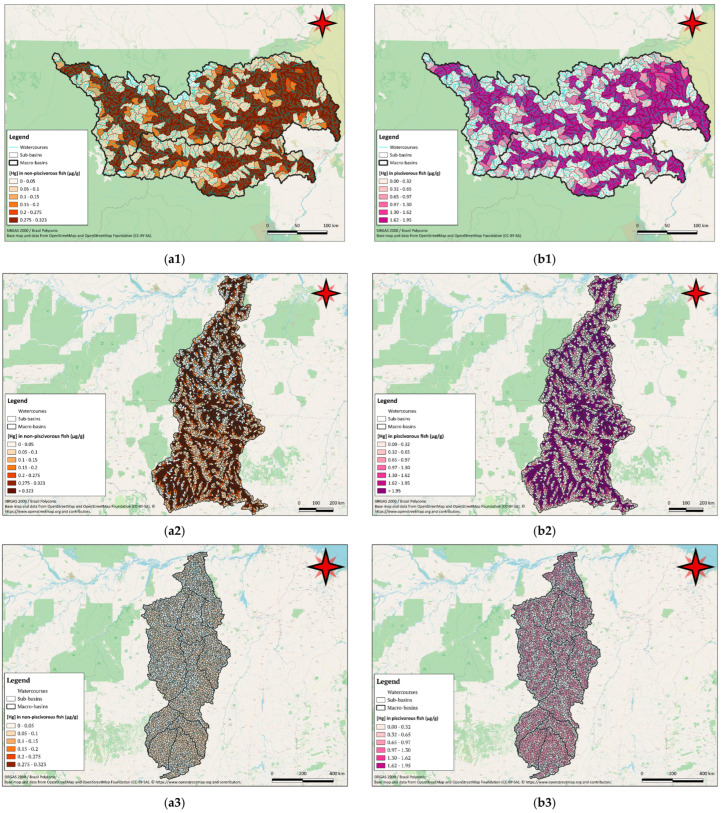
Modeled mercury concentrations in non-piscivorous (**a**) and piscivorous fish (**b**) for the Rio Branco river basin (**1**), the Tapajós river basin (**2**) and the Xingu river basin (**3**).

For all three basins, the projections of mercury bioaccumulation in fish showed lower values in the headwaters and greater accumulation in the main waterbodies, particularly in their lower reaches (Figure 3). The accumulation of mercury in fish followed the course of the rivers. The main watercourses and longer rivers showed more sub-basins with projections exceeding 0.36 µg g^−1^ for non-piscivorous fish and 6.46 µg g^−1^ for piscivorous fish in the Tapajós river and 0.092 μg g^−1^ for non-piscivorous fish and 0.7017 μg g^−1^ for piscivorous fish in the Xingu river.

### 3.2. Bioaccumulation in the Tributaries

The major tributaries were also noteworthy for their higher bioaccumulation projections. In the Uraricoera river basin, higher projected concentrations were observed in the main affluents: the Parima, Uraricaá and Amajari rivers. Similarly, in the Mucajaí river basins, notable bioaccumulation was found in the Apiaú river.

In the Tapajós river tributaries, significant projections were noted southwest of the Juruena macrobasin, including the Camararé river and its affluents, and the Teles Pires river basin. The Apiacás and Peixoto de Azevedo river basins also had high proportions of units with elevated modeled values. However, the Upper Tapajós basin showed better conditions due to the predominance of smaller watercourses. Similarly, in the Xingu river, tributaries with significant projections were noted in the southern part of the basin, including the Ronuro river and Xingu Headwaters macrobasins. In the Iriri river macrobasin, tributaries such as the Curuá, Carajari and Noo also showed elevated projections. The Lower Xingu macrobasin, with smaller watercourses, showed the best conditions.

### 3.3. Wetlands, Flooded Forests and Mining Activities

**Figure 4:**
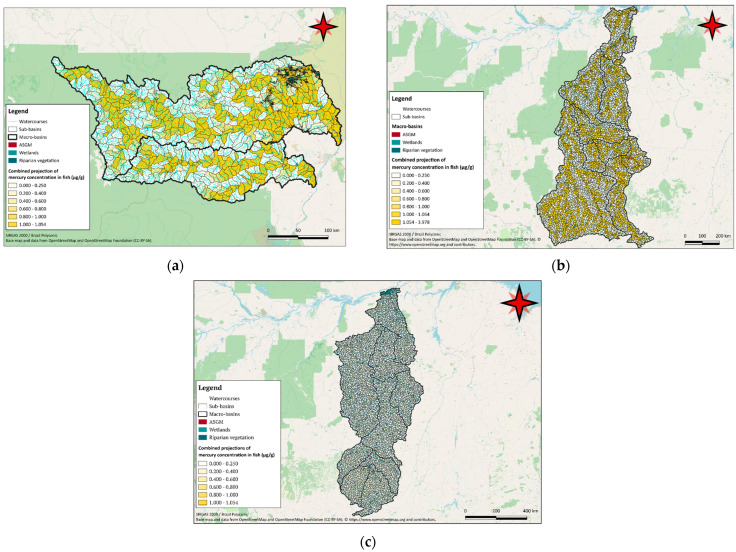
Map combining projections of mercury bioaccumulation in fish and mining site distribution, wetland and riparian areas within the Rio Branco River basin (**a**), the Tapajós river basin (**b**) and the Xingu River basin (**c**).

The projections showed a pattern of mercury accumulation increasing downstream, primarily associated with methylation dynamics and the presence of mining sites, as demonstrated in Figure 4. Other factors like water body depth and dissolved organic carbon, significantly influenced the outcomes. As the depth increases and the environment tends to become anoxic, mercury complexed to dissolved organic carbon is unlikely to settle and is subject to methylation [51].

Wetland areas are geographically associated with elevated projections of mercury concentrations in fish, even with minimal mining presence, due to increased methylation rates from baseline mercury concentrations and gas emissions [52]. Our findings show that the presence of wetlands in the northeast of the Uraricoera basin, known as Lavrado, was geographically linked to elevated mercury concentrations in fish, likely due to higher methylation rates [14].

In the Tapajós basin, wetland areas in the Upper Juruena [52] also showed elevated mercury projections in fish despite minor mining activity. The headwaters of the Xingu River, Upper Xingu and Ronuro and Suiá-Miçu river basins are rich in ecologically important floodplains [53]. The Volta Grande do Xingu in the Lower Xingu macrobasin near the Belo Monte Hydroelectric Power Plant also has abundant wetlands [54].

However, the presence of known mining activity was also visible in the results. In the Xingu basin, the Fresco river macrobasin, with its high mining activity, showed significant mercury projections in fish. In the Iriri river macrobasin, mining sites correlated with high mercury accumulations, especially in the headwaters of certain tributaries like the Curuá river.

Overall, the projections showed a consistent pattern of mercury accumulation increasing downstream, which was more associated with methylmercury dynamics than gold mining sites.

### 3.4. Compliance with Brazilian Regulation

The modeled concentration of mercury in fish for each sub-basin was compared to the Brazilian Regulation on Maximum Limits of Inorganic Contaminants in Foods [36]. The results are shown in Table 4, and the geographic distribution of each sub-basin output is available in Figure S1.

The first model for the Uraricoera and Mucajaí basins projected that 306 sub-basins, or 56.7% of the total, would not meet the maximum limits in foods based on the model’s results. Specifically, the Uraricoera river basin showed the poorest outcomes, where 57.87% of the sub-basins would fail to comply with regulations.

In the Tapajós river basin, out of the 3791 sub-basins analyzed, 1963, or 51.77%, would not meet the parameters set by Brazilian regulation [36]. Analysis of each macro-basin revealed greater non-compliance in the Lower Teles Pires basin (62.07%), Apiacás river basin (61.06%) and Upper and Middle Teles Pires basins (59.60% and 59.40%, respectively). The Juruena macrobasin showed 50.71% non-compliance, the Peixoto de Azevedo macrobasin showed 53.23% non-compliance, the Lower Tapajós basin showed 50% non-compliance, and the Jamanxim river macrobasin showed 50.71% non-compliance. The Upper Tapajós macrobasin had the least non-compliance at 43.46%.

However, in the Xingu river basin modeled results, the maximum values were capped based on the observation data from Souza-Araujo et al. (2022) [34]. The study monitored mercury concentrations in fish in the Xingu river basin and found lower average and maximum levels compared with the other basins [34]. Consequently, the statistical adjustment based on these records constrained the results to lower values, suggesting that the modeled concentrations would not exceed Brazilian regulatory limits.

### 3.5. Risk Analysis

The health risks of ingesting projected levels of bioaccumulated mercury in fish were analyzed for each basin, for each community type (riverine or indigenous) based on diet patterns and for men and women separately based on consumption. The records are shown in Table 5. The results were corroborated with the locations of indigenous villages to identify risk hot spots.

In the Uraricoera and Mucajaí basins, the riverine population risk analysis revealed that 271 sub-basins, or 50.2%, posed an extremely high risk for men. For women, all sub-basins were classified as high-risk territories due to women’s lower daily fish consumption.

The modeled results for the indigenous populations were even more concerning. For indigenous women, 276 sub-basins, or 51.1% of the assessed territorial units, were classified as extremely high-risk areas. For men, 290 sub-basins, or 53.7%, fell into the highest risk category, with other territories classified as high-risk areas.

**Figure 5:**
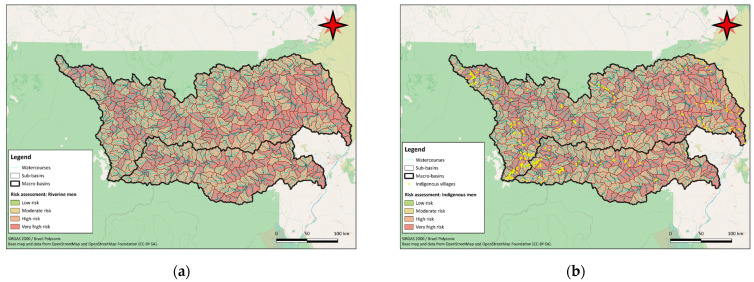
Map showing the potential risk of each sub-basin in the Branco River basin for male riverine populations (**a**) and male indigenous populations (**b**) compared with the locations of indigenous villages, based on the model’s projected results and risk categories.

A comparison with the locations of indigenous villages provided by the Instituto Socioambiental—Programa Rio Negro (2023) highlights a significant risk for traditional communities in the upper Mucajaí, Parima and Auaris river catchments to the west of the basins. There are also relevant risks in the lower Uraricoera and Parimé rivers, influenced by the wetland areas of the Lavrado region. The geographic representation of the risk for male riverine and indigenous populations, who are more exposed to mercury due to higher fish consumption, can be seen in Figure 5. The results for the female populations are available in Figure S2.

**Figure 6:**
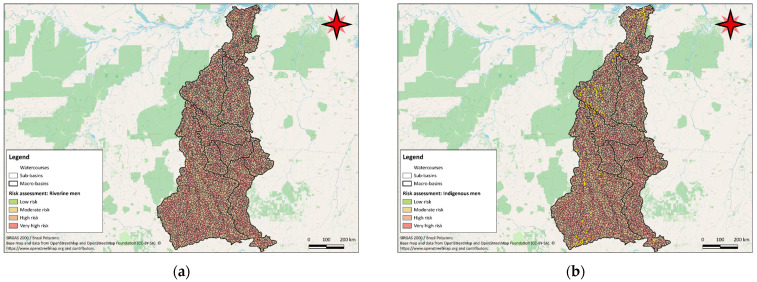
Map showing the potential risk of each sub-basin in the Tapajós River basin for male riverine populations (**a**) and male indigenous populations (**b**) compared with the locations of indigenous villages, based on the model’s projected results and risk categories.

In the Tapajós River basin, risk analysis for the riverine populations indicated that 1711 sub-basins, or 45.1%, posed an extremely high risk for women. For men, 1871 sub-basins, or 49.4%, were classified as extremely high risk due to higher fish consumption. The geographic representation of the risk for male riverine and indigenous populations, who are more exposed to mercury due to higher fish consumption, can be seen in Figure 6. The results for the female populations are available in Figure S3.

For the indigenous populations, 1879 sub-basins, or 49.6%, were extremely high-risk areas for women, and 1912 sub-basins, or 50.4%, were classified as such for men. A comparison with indigenous village locations provided by the National Foundation for Indigenous Peoples highlighted significant risks in the Upper and Middle Juruena, particularly in the Lower Teles Pires, where higher mercury accumulation in fish coincides with higher concentration of Indigenous communities.

In the Xingu basin model, the limitation of maximum values in the model, based on the work of Souza-Araujo et al. (2022), resulted in projections which varied within a narrow range of concentrations [34]. Consequently, all results fell into the high-risk category when conducting the risk analysis. The representation in map form is available in the Supplementary Material in Figure S4.

These findings provide an important insight. While the adjusted results did not exceed Brazilian regulatory limits, all basins fell into the high-risk category, capable of inducing harmful effects to the nervous system. This discrepancy arises from the fact that the maximum mercury limits in foods under Brazilian legislation do not take into account the high fish consumption by Amazonian populations.

Although the projections ranged from 0.13 μg kg^−1^ bw^−1^ d^−1^ for women living in less-affected sub-basins to 1.02 μg kg^−1^ bw^−1^ d^−1^ for indigenous men from the most affected territories, the results consistently fell into the high-risk category. Despite the uniform risk classification, the pattern reveals important aspects of contamination, such as the higher risks faced by men compared with women and the increased vulnerability of indigenous communities due to higher fish intake.

## 4. Discussion

The study projections revealed a pattern; the mercury concentrations in fish exhibited an ascending trend downstream within basins. In the upper-tier stream results, both non-piscivorous and piscivorous fish showed higher average mercury levels, indicating a greater susceptibility to mercury bioaccumulation in mid- and lower-course rivers. While acknowledging the model’s limitations due to the scarcity of sampling information, identifying this pattern provides valuable insights which can help overcome regional data gaps and enhance strategies for effective mercury contamination control.

This study’s limitations notwithstanding, this observed pattern is consistent with the findings of Carrasco et al. (2011), who observed that the geometric mean concentrations of total mercury and methylmercury in fish consistently increased downstream along a 90 km section of the Spanish Ebro River, starting from the contamination source [55]. Similarly, Carrasco et al. (2008) reported elevated mercury levels over 10 km downstream from the contamination source on the same watercourse [56].

Our results also align with the observations from Ward, Nislow and Folt (2010), who discussed the significant role of methylmercury production within streams in driving mercury bioaccumulation, rather than merely the presence of contamination sources [57]. In our projections, ascending mercury concentrations in upstream areas appeared to not only be related to the density of contaminating gold mining sites within the sub-basin but also mercury methylation dynamics.

The results, especially in the Xingu river basin, underscore how mine locations determine metal accumulation in certain areas. Additionally, the territorial distribution of wetlands plays a crucial role in mercury bioaccumulation [16]. Areas such as the Lavrado, Upper Juruena wetlands and Upper Xingu floodplains exhibited higher mercury bioaccumulation in fish due to potential methylmercury transformations taking place in the regions. The biogeochemical conditions in wetlands, such as a high organic matter content and oxygen deficiency, favor the methylation process and mercury bioaccumulation [51].

Projections indicate that 27.47% of the analyzed sub-basins would not meet the current Brazilian legislative parameters for mercury in fish. It is important to note that although 72.53% of the basins comply with legal parameters, this result is significantly influenced by the modelling process of the Xingu River basin. After statistical adjustment based on the sampled study data, 100% of the basins fell within the maximum limits. When focusing solely on the Branco and Tapajós river basins, 52.38% of the sub-basins would potentially exceed the maximum mercury limits for fish, highlighting the importance of a regional analysis.

The risk assessment results are even more concerning. Given the elevated regional fish consumption rates, traditional communities in 100% of the analyzed sub-basins are potentially at high or extremely high risk. When considering only the data from the Branco and Tapajós river basins, the situation worsens. In these areas, where populations face the highest risks, at least 49.79% of the indigenous population could be subjected to severe acute kidney and liver damage.

The discrepancy between the fixed thresholds and actual risk arises from differing evaluation approaches. Brazilian regulatory limits, established in 1998, set fixed standards for mercury levels in fish at 0.5 μg g^−1^ for non-piscivorous and 1.0 μg g^−1^ for piscivorous fish [36]. While such thresholds have been adopted globally, agencies like the US Food and Drug Administration (FDA) and WHO have shifted toward standards based on the daily mercury intake per body weight [58,59].

Fish consumption in the Amazon, especially within traditional communities, ranks among the highest in the world [33]. Therefore, relying solely on fixed thresholds or restricting fish consumption may not effectively mitigate mercury contamination in the region. Despite limited available information in this socially diverse area, encompassing large cities, various ecosystems and a myriad of traditional communities, the findings, even with their constraints, suggest that Brazil requires regulatory revisions to enhance contamination control and management. Adopting a tailored approach grounded in specific risk assessments which incorporate local data on fish consumption rates and monitoring information could ensure broader protection while respecting dietary practices across different contexts.

### Limitations

A key challenge stems from the limited available environmental information. Relying on previous studies from the analyzed river basins rather than collected field data may affect precision. Obtaining region-specific information would improve the model’s efficiency. Additional standardized field data sampling could help calibrate the framework to produce more realistic scenarios.

While SERAFM generally does not require calibration, adjusting the model rate constants can enhance projection accuracy [16]. Sensitivity analysis and Monte Carlo simulations are recommended for such calibration [18]. However, due to limited comprehensive environmental data, calibration was not feasible in this study. Targeted monitoring would contribute to validating the results and generating more accurate outputs [17].

Improving input data quality is also crucial for obtaining even more reliable results from a statistical model. While this work focused on modeling large areas to assess and compare conditions across basins, detailed local data could offer more representative projections. Field monitoring and data collection would facilitate model adjustments based on environmental observations, though obtaining and analyzing field samples remains challenging at the Amazon scale. Enhanced monitoring and data collection are necessary to refine outputs and adjust the assumptions used in the modeling process.

This study did not account for occupational mercury exposure, which is a relevant pathway for mercury exposure in gold mining sites. Mercury health effects often result from combined occupational and dietary exposure, primarily affecting men who inhale mercury vapors during gold amalgamation [10].

Finally, this study did not address broader ecological impacts of mercury bioaccumulation. Mercury disrupts food chains and affects fish predators, engendering cascading effects [10]. Further research should explore ecological implications and develop strategies to mitigate mercury contamination impacts on Amazonian ecosystems.

## 5. Conclusions

This study analyzed watersheds representing 27% of the Brazilian Amazonian territory, areas profoundly impacted by non-industrial gold mining. Despite challenges in representing such a vast and diverse area, SERAFM demonstrated potential in providing insights into mercury distribution and bioaccumulation in the region, overcoming data scarcity. The modeled results highlighted the pattern of mercury accumulation downstream in the basins. Another crucial finding is the influence of wetlands and riparian areas on methylmercury transformation and subsequent fish accumulation. Notably, regions such as the Lavrado in Roraima and the wetlands and floodplains in the Upper Juruena and Upper Xingu regions markedly contributed to increased projections despite the mining locations.

Furthermore, risk assessments revealed an urgent need to review Brazilian regulations for mercury in fish. According to projections, 27.47% of the analyzed sub-basins would not comply with the Brazilian Regulation on Maximum Limits of Inorganic Contaminants in Foods. These results raise concerns about mercury contamination. However, risk assessments, considering the high fish consumption in the region, especially by traditional communities, indicated a more alarming scenario; at least 26% and 26.6% of the riverine and indigenous populations, respectively, are at extremely high risk.

The situation becomes even more critical when weighing the outputs from the Xingu river basin, where statistical adjustment places the entire territory in the high-risk category. In 33.48% of the analyzed sub-basins from the Branco and Tapajós river basins combined, mercury poses an extremely high risk to riverine populations, while in 49.79%, it poses an extremely high risk to indigenous populations.

While this study provides a relevant perspective, it is important to acknowledge its limitations. The data used were compiled from various sources, and projections rely heavily on scarce existing data, which might not fully capture regional variations due to the vastness of the area. These limitations highlight the need for improved data collection and monitoring in the Amazon to refine and validate the model further. Additionally, incorporating new mercury data with a more representative spatial distribution could address potential biases caused by data gaps. Socioeconomic information is also highly important and can improve risk assessments.

To enhance the accuracy and reliability of our findings, future studies should conduct more comprehensive analyses considering direct environmental sampling and laboratory measurements. An integrative initiative based on collaboration between the governments and academic institutions of Amazonian countries could help develop and implement national plans for monitoring mercury contamination. Generating and consolidating more information through monitoring programs would enable specialists to better understand mercury behavior in such a complex environment.

There is also an urgent need to adjust the Brazilian Regulation on Maximum Limits of Inorganic Contaminants in Foods to the regional context, taking into account local food habits and other exposure pathways. The severity of mercury contamination in fisheries within Amazonian countries calls for federal regulatory agencies to revise the regulatory limits for fish in areas potentially affected by gold mining, thereby reducing contamination risks for populations consuming these fish.

Finally, all addressing initiatives must respect Amazonian traditions. To control the dietary mercury intake by the local population, it is essential to create advisory systems tailored to each context, respecting cultural and behavioral practices alongside the environmental aspects. The Amazon is a diverse territory biologically and socially, with characteristics which must be respected and preserved. While general models need to broaden perspectives to compare scenarios, specific approaches must consider each region’s unique realities.

## Figures and Tables

**Table 1:** Average body weight in northern Brazilian populations. The 95th percentile values determined for non-piscivorous and piscivorous fish.

River Basin	Dataset	95th Percentile	Reference
Branco River	Non-piscivorous fish	0.3233 µg g^−1^	[33] De Vasconcellos et al., 2022
	Piscivorous fish	1.9465 µg g^−1^	
Tapajós River	Non-piscivorous fish	0.3566 µg g^−1^	[30] The Mercury Observatory, 2023
	Piscivorous fish	6.4600 µg g^−1^	
Xingu River	Non-piscivorous fish	0.0920 µg g^−1^	[34] Souza-Araujo et al., 2022
	Piscivorous fish	0.7017 µg g^−1^	

**Table 2:** Average body weight in northern Brazilian populations.

Gender	Average Body Weight (kg)
Men	78.2
Women	66.9

**Table 3:** Fish consumption among traditional communities.

Community	Gender	Daily Non-Piscivorous Fish Consumption (g)	Daily Piscivorous Fish Consumption (g)	Daily Total Fish Consumption (g)
Riverine	Male	189.7	85.37	104.33
	Female	124	55.8	68.2
Indigenous	Male	216.75	97.54	119.21
	Female	168.58	75.86	92.72

**Table 4:** Compliance and non-compliance rates with the Brazilian Regulation on Maximum Limits of Inorganic Contaminants in Foods for the projected mercury concentration in fish in each basin.

River Basin	Compliance	Non-Compliance
Branco River	43.33%	56.67%
Tapajós River	48.23%	51.77%
Xingu River	100.00%	0.00%
Total	72.53%	27.47%

**Table 5:** Compliance and non-compliance rates with Brazilian safety standards for the projected mercury concentration in fish in each basin.

River Basin	Traditional Community	Gender	Risk Categories		
			Moderate Risk	High Risk	Extremely High Risk
Branco River	Riverine community	Men	0.0%	49.8%	50.2%
		Women	0.0%	100.0%	0.0%
	Indigenous community	Men	0.0%	46.3%	53.7%
		Women	0.0%	48.9%	51.1%
Tapajós River	Riverine community	Men	0.0%	50.6%	49.4%
		Women	0.1%	54.8%	45.1%
	Indigenous community	Men	0.0%	49.6%	50.4%
		Women	0.0%	50.4%	49.6%
Xingu River	Riverine community	Men	0.0%	100.0%	0.0%
		Women	0.0%	100.0%	0.0%
	Indigenous community	Men	0.0%	100.0%	0.0%
		Women	0.0%	100.0%	0.0%
Total	Riverine community	Men	0.0%	74.0%	26.0%
		Women	0.0%	79.3%	20.7%
	Indigenous community	Men	0.0%	73.4%	26.6%
		Women	0.0%	73.9%	26.1%

## Data Availability

Data are contained within the article or Supplementary Material, and further datasets are available on request from the authors.

## Supplementary Materials

The following supporting information can be downloaded at https://www.mdpi.com/article/10.3390/toxics12080599/s1, Figure S1: Sub-basins in which the modeled mercury concentrations in fish exceed the Brazilian Regulation on Maximum Limits of Inorganic Contaminants in Foods within the Rio Branco river basin (a), the Tapajós River basin (b) and the Xingu river basin (c). Figure S2: Map showing the potential risk of each sub-basin in the Branco river basin for female riverine populations (a) and female indigenous populations compared with the locations of indigenous villages, based on the model’s projected results and risk categories. Figure S3: Map showing the potential risk of each sub-basin in the Tapajós river basin for female riverine populations (a) and female indigenous populations compared with the locations of indigenous villages, based on the model’s projected results and risk categories. Figure S4: Map showing the potential risk of each sub-basin in the Xingu river basin for male and female riverine or indigenous populations, based on the model’s projected results and risk categories. Sheet S1: Dataset of input parameters for mercury fate and transport modeling using SERAFM for every assessed sub-basin. Sheet S2: Modeled methylmercury bioaccumulation in non-piscivorous and piscivorous fish in each sub-basin of the assessed watersheds estimated using the SERAFM framework.

## Appendix A

**Input Parameter**	**Database**
Watershed location	Rainfall regimes significantly and positively influence soil erosion [60]. Therefore, the input data were defined as “east”, since the equation was designed for American climatic conditions [16]. The model references “east” as the eastern portion of the American territory east of the Mississippi River, which has high rainfall patterns like those of the Amazon region [61]. The same strategy was adopted by Agurto (2012) when projecting the dynamics of mercury in the Peruvian Amazon [18].
Watershed area	The sub-basins and their characteristics were defined using the HydroBASINS database [15].
Percent impervious	For determining the impervious portion of each sub-basin unit, MapBiomas Collection 8 was employed to assess the urban areas in the analyzed territory [62].
Percent wetland	For determining the wetlands portion of each sub-basin unit, MapBiomas Collection 8 was employed to assess the wetlands in the analyzed territory [62].
Percent riparian	For determining the riparian portion of each sub-basin unit, MapBiomas Collection 8 was employed to assess the flooded forests in the analyzed territory [62].
Percent with known contaminated soil	The portion of contaminated soil was determined by considering the mining areas identified by the MapBiomas Collection 8 database [62]. To establish each territory, a buffer zone was delineated around the mining sites. Technical guidelines for mercury monitoring in the environment suggest that the highest mercury concentrations are found within 500 m of mining areas [63].
Lake area	The sub-basins and their characteristics were defined using the HydroBASINS database [15].
Epilimnion depth	The sub-basins and their characteristics were defined using the HydroBASINS database [15]. Epilimnion depth was obtained by deducing 0.1 m from the total depth.
Hypolimnion depth	In lotic systems, the model recommends a hypolimnion layer of 0.1 m [16].
Anoxic hypolimnion	In homogeneous lotic systems, such as smaller rivers, the model recommends not considering an anoxic hypolimnion layer and setting the input data to “no” [16].
Hydraulic residence time	The hydraulic retention time of each analyzed sub-basin was calculated by dividing the volume of the water body by its discharge. Both pieces of information were obtained from the HydroBASINS database [15].
Water pH	The National Water Agency has various monitoring stations distributed across the country’s water bodies. To obtain the water’s pH level, the average of the records from the best-located station was used in the modeling, usually in groups of nearby sub-basins [64].
Epilimnion water temperature	The National Water Agency has various monitoring stations distributed across the country’s water bodies. To obtain the epilimnion water temperature, the average of the records from the best-located station was used in the modeling, usually in groups of nearby sub-basins [64].
Air temperature	The air temperature in each sub-basin was obtained from the WorldClim database [65] using historical climate data processed using the RASTER Calculator tool in the QGIS application [22].
Annual precipitation	The annual precipitation in each sub-basin was obtained from the WorldClim database [65] using historical climate data processed using the RASTER Calculator tool in the QGIS application [22].
DOC epilimnion	A study conducted by Belger and Forsberg (2006) showed that the dissolved organic carbon concentrations reached 2.99 mg L^−1^ in the Branco River [66]. For the modeling, the results obtained were extrapolated to the Uraricoera and Mucajaí rivers, since their characteristics are similar [67]. Studies conducted in the Tapajós River basin were used to determine dissolved organic carbon in the regional watercourses, ranging from 2.22 [68] to 3.8 mg L^−1^ [69]. Guedes (2020) studied the carbon dynamics in different watercourses of the Amazon basin and found an average dissolved organic carbon concentration in the Rio Xingu of 3.0 ± 0.5 mg L^−1^, ranging from 2.2 mg L^−1^ at the lowest flow to 3.1 mg L^−1^ at the highest flow. This study was used in the basin modeling [70].
DOC hypolimnion	
Inflow mercury concentration: Hg0	The SERAFM model considers the pattern of mercury concentrations in the input flow of the system as null [16]. As the protocol will be used to calculate metal levels in upstream sub-basins, the results will be used as inputs in subsequent modeling.
Inflow mercury concentration: HgII	
Inflow mercury concentration: MeHg	
Total mercury concentration in contaminated sediment (dry weight)	Jacques et al. (2023) studied samples from the Rio Mucajaí and found average concentrations of 0.132 µg g^−1^ of metal in the sediment [71]. These values were the only ones found for the basins of the Uraricoera and Mucajaí rivers and were used in the modeling. Lino et al. (2019) analyzed the mercury concentrations in different matrices along the Tapajós River basin and found results ranging from 0.019 to 0.155 µg g^−1^ in sediment samples. The average of 0.074 ± 0.032 µg g^−1^ obtained in the study for the 27 samples analyzed will be used in the modeling [72]. Araujo et al. (2018) studied mercury speciation in water bodies of the Amazon basin and found an average of 0.939 μg g^−1^ of metal in the two samples collected from the Rio Xingu. The average concentration of monomethylmercury found was 0.02585 μg g^−1^ [73].
Known mercury in contaminated soils: Cs, Hg0	Egler et al. (2006) studied a gold mining site along thte Creporizinho River and found average concentrations of mercury of 0.94 µg g^−1^ in the finer soil particles, which are more easily eroded [74]. These values were used in the modeling, considering that the mining areas correspond, in the methodology, to contaminated areas. The mercury concentrations in sediment and contaminated soils will be obtained from the scientific literature. Previous studies conducted in Brazil have shown that contaminated soils typically contain a mixture of elemental mercury and its oxidized species [75,76]. Therefore, general data indicating total mercury concentrations were equally partitioned between “Cs,Hg0” and “Cs,HgII”. Soil density will be considered in the conversion.
