# Supplementary material for: Mercury Dynamics and Bioaccumulation Risk Assessment in Three Gold Mining-Impacted Amazon River Basins
Source: Toxics. 2024 Aug 18;12(8):599. doi: 10.3390/toxics12080599 (PMC11359172; doi:10.3390/toxics12080599)
Supplement: Supplementary file 1 [file toxics-12-00599-s001.zip › Article - Supplementary Material.pdf]

toxics

MDPI

Article

The Downstream Dynamics and Risk Assessment of Mercury Bioaccumulation in three Brazilian Amazon River Basins Affected by Gold Mining

Vitor Domingues, Carlos Colmenero, Maria Vinograd, Rodrigo Balbueno and Marcelo Oliveira

Supplementary Material

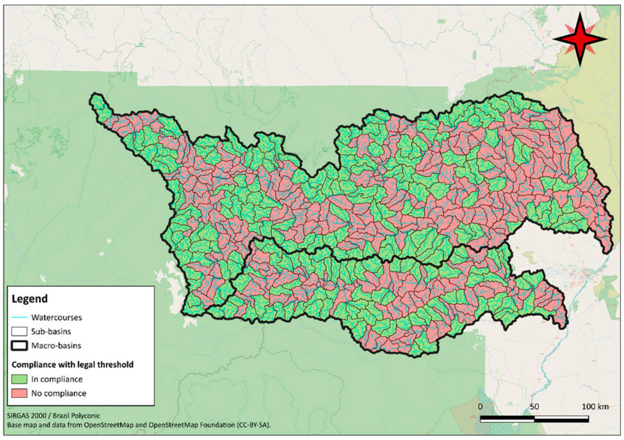

(**a**)

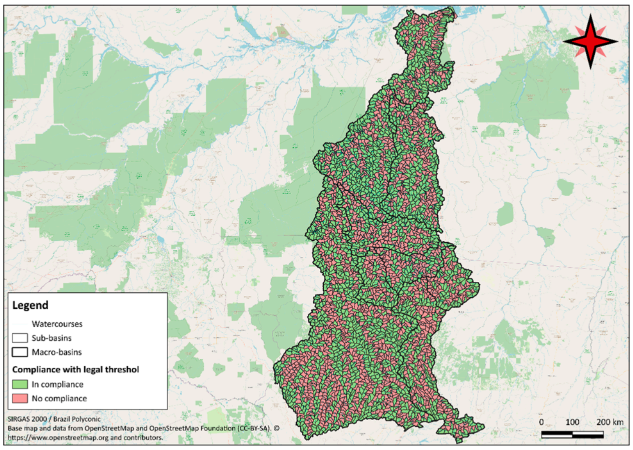

(**b**)

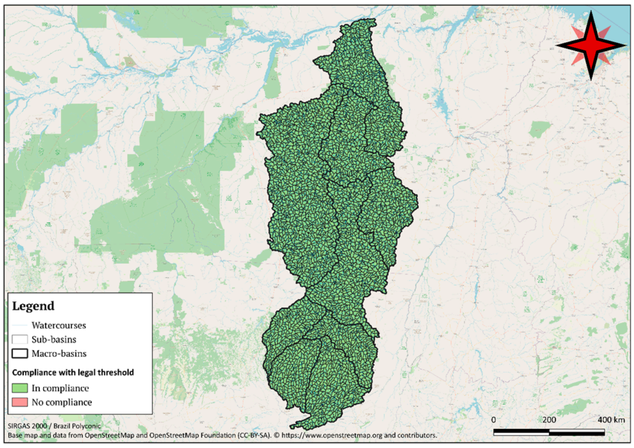

(**c**)

Figure S1. Sub-basins in which the modelled mercury concentrations in fish exceed Brazilian Regulation on Maximum Limits of Inorganic Contaminants in Foods within the Rio Branco River Basin (a), the Tapajós River Basin (b), and the Xingu River Basin (c).

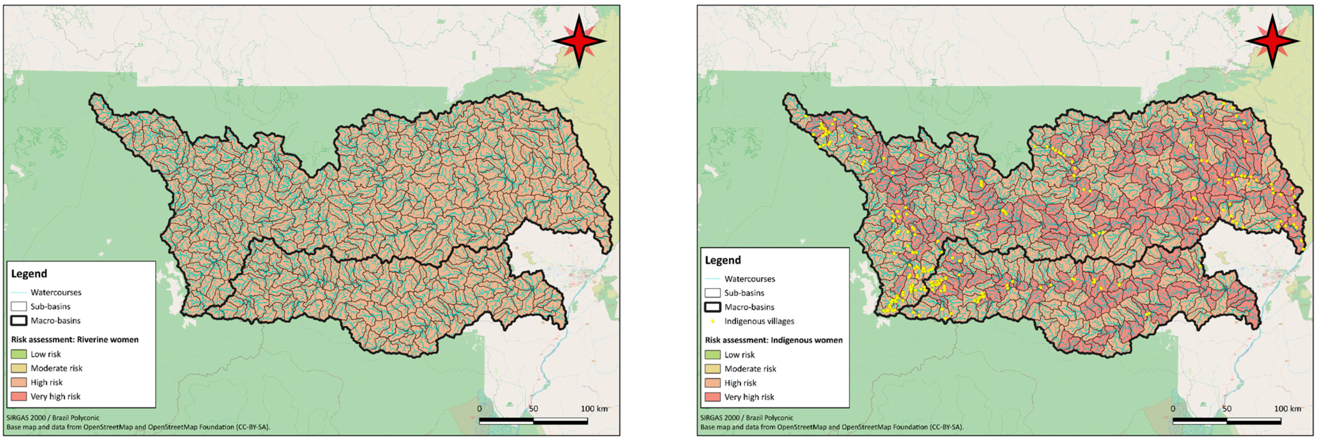

(**a**) (**b**)

Figure S2. Map showing the potential risk of each sub-basin in the Branco River Basin for female riverine populations (a) and female indigenous populations compared with the location of indigenous villages, based on the model's projected results and risk categories.

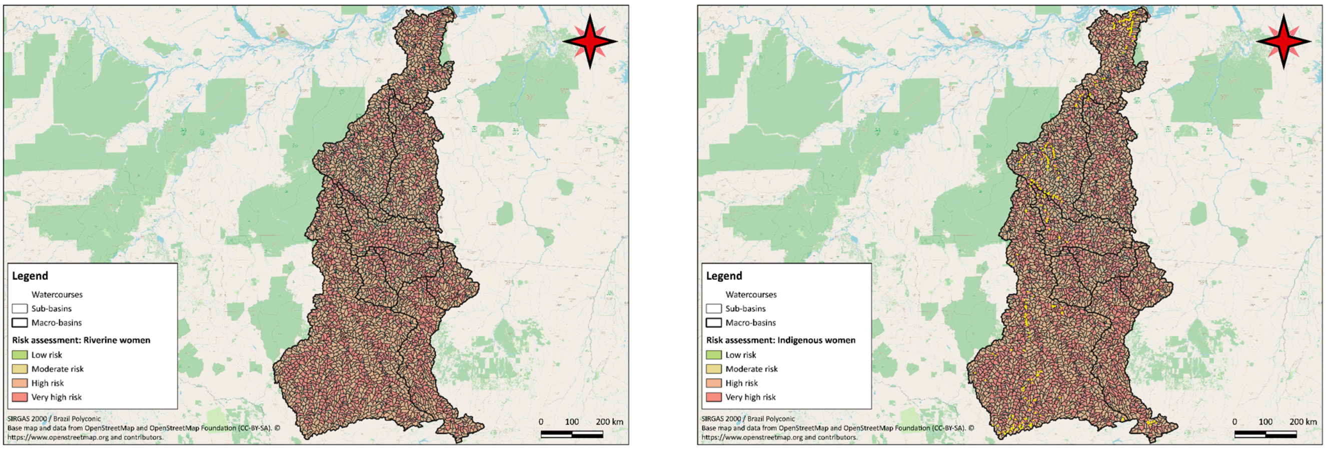

(**a**) (**b**)

Figure S3. Map showing the potential risk of each sub-basin in the Tapajós River Basin for female riverine populations (a) and female indigenous populations compared with the location of indigenous villages, based on the model's projected results and risk categories.

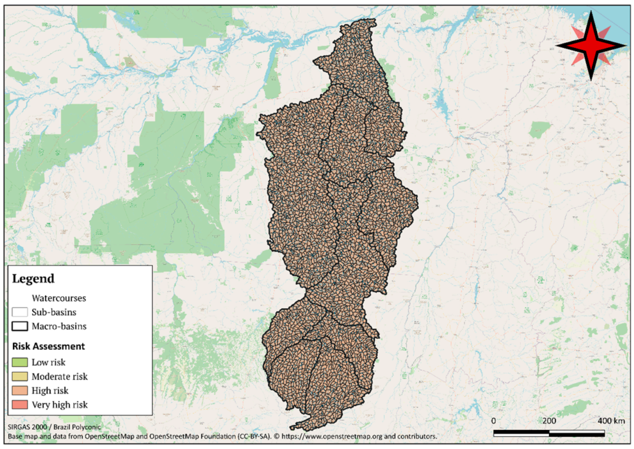

Figure S4. Map showing the potential risk of each sub-basin in the Xingu River Basin for male and female riverine or indigenous populations, based on the model's projected results and risk categories.
